# Fatal Pericarditis, Caused by Lodgment of Artifical Teeth and Gold Plate in the Œsophagus

**Published:** 1858-04

**Authors:** J. R. Buist

**Affiliations:** House Physician, Bellevue Hospital, N. Y.


### ARTICLE XI.

*Fatal Pericarditis, caused by lodgment of Artificial Teeth and Gold Plate in the Œsophagus.* Reported by J. R. Buist, M. D., House Physician, Bellevue Hospital, N. Y.

D. M———, a Scotchman, aged forty, of full muscular development, but of very intemperate habits, was admitted to Bellevue Hospital, October 1st, under the supervision of Dr. A. Clark.

His account of himself was, that on the day previous he had accidentally swallowed two of his teeth, which were then in his stomach, occasioning some pain and very uneasy sensations, in consequence of which he could neither eat nor sleep. The man, however, confessed to have been just indulging in a long debauch, and was apparently on the verge of delirium tremens; so his story was regarded as a mere whim. Besides, the teeth were supposed to be natural ones, and if they should be in the stomach, it was thought that they would occasion no inconvenience. The patient was therefore allowed milk, teas, &c., and left to time. In a few days he seemed much better; the nervous tremor had subsided, he had slept some, and at his own request was discharged October 5th.

On the 10th he re-entered the hospital, with the symptoms at first complained of much aggravated, and persisting in his story of having swallowed his teeth. There was severe pain at the epigastrium, extending around the left side to the spinal column, accompanied with constant nausea, a disgust for all food, and an entire loss of sleep. His countenance was anxious, and of a dusky paleness; tongue coated white, and moist; skin natural; respiration a little accelerated, and the pulse in the neighborhood of 100, very small and weak. There was no marked tenderness over the chest or abdomen. On being closely questioned, he made it known, for the first time, that the *teeth* he had swallowed were artificial ones, set on gold plate, all of which had slipped down his throat while eating some soup. All that was given to him in the way of medicine or nourishment, was immediately rejected. No improvement in his condition occurred after his admission. His pulse and respiration were not at any time of marked frequency. Some thirty hours before death he became delirious; his face was of a livid hue, and a cold sweat covered the whole body.

Death occurred at 9 A. M., October 14th. On inspection of the body, thirty hours after death, the stomach and intestinal tract were found healthy, with the exception of some congestion near the pyloric orifice of the former, and a superficial ulcer, probably post mortem, at the greater cul-de-sac. On removing the sternum, the pericardium was seen to be greatly distended, thickened, and inflamed. Through an accidental opening a quantity of gas escaped, of a disagreeable, gangrenous odor. On laying the pericardium fairly open, several ounces of seemingly sero-purulent fluid, of a dark greenish color, was seen; both of its surfaces were in parts disorganized; in other parts covered with a thick coating of lymph. Examining further into the adjoining tissues, a foreign body was discovered in the œsophagus, two and a half inches above the stomach, which had ulcerated through into the right and posterior part of the pericardium. This proved to be the teeth and a gold plate, the latter about one and a half or two inches long, and one inch broad.

The lungs were congested to the last degree; other organs not examined.

The specimen is now preserved in the museum of the hospital.—*Charleston Medical Journal and Review.*

